# Cs-137 in the natural environment of the Gorce Mountains (Poland)

**DOI:** 10.1007/s10967-014-3144-8

**Published:** 2014-04-29

**Authors:** Paweł Jodłowski, Chau Nguyen Dinh

**Affiliations:** Faculty of Physics and Applied Computer Science, AGH University of Science and Technology, Al. Mickiewicza 30, 30-059 Kraków, Poland

**Keywords:** Cs-137, Gorce Mts., Soil, Lichens, Chernobyl, Gamma-spectrometry

## Abstract

Concentration of activity of Cs-137 and its spatial distribution in soils and lichen *Hypogymnia physodes* were determined in the Gorce Mts. (several hundred km^2^) in S Poland. The authors distinguished two areas of the Gorce Mts. on the basis of markedly different Cs-137 depositions, whose respective average values are 4.4 and 9.9 kBq/m^2^ as at 1st July 2005. The average Cs-137 activity concentration in the lichen *H*. *physodes* from the Gorce is 47 Bq/kg d.m. A significant local variability of quantities measured amounts to a few dozen percent was found.

## Introduction

The authors have attempted to measure the activity concentration of Cs-137 in the natural environment of the Gorce Mts. (Poland). The distribution of contamination by radioactive Cs-137 in Poland (South Poland in particular) (cf. section Deposition of Cs-137 and [1, 10–13]) and in its immediate vicinity (e.g. [2, 3]) is well known, but there is a lack of detailed data pertaining to the level and distribution of this contamination in smaller areas, covering only several hundred square kilometres. Such investigations provide, on the one hand, information on the current distribution of the activity concentration, and on the other hand, may establish a point of reference in the future when dealing with the changes of this distribution in the course of time. Moreover, baseline studies of this type are necessary because of the location of nuclear power plants in the immediate vicinity of Poland and the anticipated construction of such a plant in Poland around the year 2025.

**Fig. 1:**
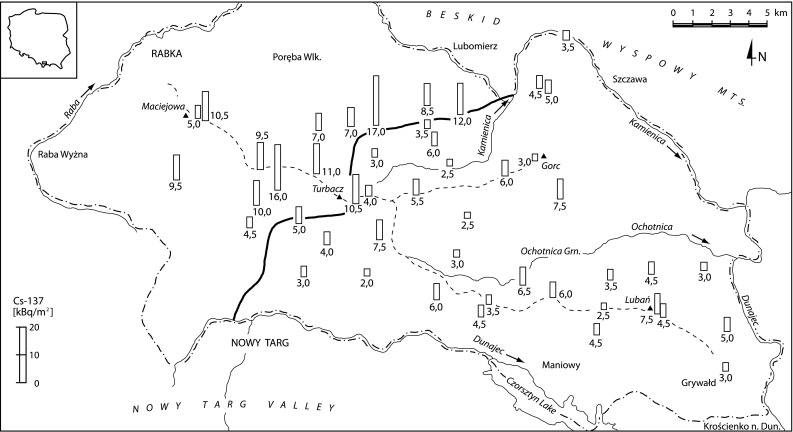
Deposition of Cs-137 in the selected sites of the Gorce Mts. The Cs-137 deposition values have been presented in numerical and graphic form and rounded up to 0.5 kBq/m^2^. The border shown on the map (*solid bold line*) separates two areas of different Cs-137 deposition

The Gorce Mts. situated in Southern Poland (Fig. 1) form part of the Western Beskidy Mts., approximately 44 km in length, a width of approximately 15 km and an area of approximately 530 km^2^. The highest peak is Mt. Turbacz (1,310 m asl). In 1981, the central and north-eastern parts of the Gorce Mts. were declared the Gorce National Park, which presently has an area of around 70 km^2^. The geological structure of the Gorce Mts. is made of folded and thrusted flysch formations: mainly the Magura quartz sandstones cemented with silica (silicon oxide) or calcite (calcium carbonate), minor conglomerates and shales. The soils of the study area belong mainly to various brown soil sub-types, in the lower subalpine zone and various podzoilic soil sub-types in the upper subalpine zone. Small areas are covered by lithogenic soils and a variety of hydrogenic soils.

The Gorce Mts. have been selected for the study as it is an area which has been practically unaffected by human economic activity, it is reflected, among others, in the presence of an intact surface soil layer and low level of contamination of the natural environment. Additionally, as a large part of the study area is located within the Gorce National Park, it is most probable that this part of the Gorce Mts. will not be significantly affected by human economic activity in the future. Therefore, it is a very good testing ground for long-term measurements of the dynamics of radioactive contamination.

## Materials and methods

The study included:

- soil;
- lichens, because this species is commonly used as bioindicators of air pollution (cf. [4]). The lichen *Hypogymnia physodes*, often used in environmental studies, was chosen in our measurements.

Sampling was carried out in the years 2001–2005 over the whole area of the Gorce Mts., in sites of dense forest complexes to minimise interference by human activity. In particular, the selected sampling sites differed in their absolute altitude (between 530 and 1,250 m asl.), slope inclination, morphology (valleys, slopes, ridges, etc.), and so on. They had to be representative of a study area and to cover it with possible regularity. A total of 74 samples of soil (49 samples) and lichen (25 samples) were collected.

Soil samples were taken from the top 10-cm layer in the form of cores with a diameter of 10 cm and a length of 10.0 cm (uncertainty 0.5 cm), using a PVC cutter. The core was next divided into three layers, according to the soil horizon types distinguished (organic, mineral). The thickness of each layer was usually 3–4 cm. The samples were further prepared for radiometric measurements in accordance with IAEA recommendations [5], applicable to environmental samples. Larger rock fragments, exceeding a diameter of 3 mm, were discarded, the remaining material was dried to a constant weight at 105 °C and ground to a grain size below 1 mm. The ground material was placed in cylindrical measuring vessels with a volume of 121 cm^3^, the activity concentration of Cs-137 (Bq/kg) in the soil samples (layers) distinguished was measured, and the Cs-137 deposition per 1 m^2^ (its activity concentration in the top 10-cm soil layer; kBq/m^2^) calculated.

The lichen samples were collected from the bark of European beech and Norwegian spruce trees at a height of between 0.5 and 1.5 m above the soil surface. This procedure should minimise the effect of dependence of the Cs-137 concentration upon the height of the sampling site (cf. [6]). The area in which samples were taken did not exceed 1 ha, while the mass of lichen samples ranged between single grams and several tens of grams. In the laboratory the lichens were identified once again and their selection was repeated. The next stage of cleaning was carried out under a magnifying glass and involved final removal of other lichen species and splitting the thallus of the lichen from the tree bark. For lichens the material collected was dried to a constant mass at around 70 °C, and next, depending on the amount of the material, either ground to a grain size below 1 mm, or broken up by hand into fragments below 5 mm. The material was placed in cylindrical measuring vessels of a volume of 48 or 121 cm^3^.

The activity concentration was determined using a semiconductor HPGe detector (Canberra GX4020) with 42 % relative efficiency and resolution of 1.9 keV, placed in lead housing with walls 10 cm thick. The standard solution of gamma emitting radionuclides, was used as a calibration source; the uncertainty of radionuclide activities should not exceed 2 %. The duration of measurements was generally chosen so that the relative uncertainty of the net count for 662 keV line (Cs-137) was less than 3 %. In further calculation the differences in the densities of sample and calibration source were considered. A detailed description of the methodology is set out elsewhere [7, 8].

## Results and discussion

The activity concentrations of Cs-137 originating from global fallout and the “Chernobyl fallout” were decay corrected to 1st July 2005. Results for all sampling sites are presented in Table 1; comprehensive results are contained in the final report of the study [9].

**Table 1:** Results of the Cs-137 analysis in the Gorce Mts.: description of sampling sites, Cs-137 deposition, Cs-137 activity concentration in the soil and lichen *H*. *physodes*

Site no	Site location^a^	Coordinates^b^	Altitude (m asl)	Gorce Mts. part^c^	Cs-137 deposition (kBq/m^2^)^d^	Cs-137 activity concentration (Bq/kg d.m.)				
						Soil layer^e^				Lichen^f^
						1st	2nd	3rd	10 cm	
1	Maciejowa (f)	49°34′55″N 20°00′35″E	830	NW	10.5	375	569	101	314	43.0
1a	Maciejowa (g)	49°34′58″N 20°00′07″E	830	NW	5.11	85	86	90	87	
2	Rdzawka Ponice (f)	49°33′12″N 19°59′21″E	770	NW	9.41	259	259	82	142	
3	Stare Wierchy (f)	49°33′44″N 20°02′56″E	1,030	NW	9.67	532	388	27	210	41.2
5	Średni Wierch (f)	49°32′44″N 20°04′20″E	1,110	NW	15.8	370	848	412	511	
6	Koninki/valley (f)	49°34′42″N 20°05′22″E	700	NW	7.04	198	118	52	104	
8	Kocurka Glade (f)	49°33′23″N 20°05′17″E	1,080	NW	11.0	251	676	114	306	
9	Turbacz (f)	49°32′36″N 20°07′12″E	1,240	NW	13.3	393	947	243	539	
9	Turbacz (f)	49°32′32″N 20°07′17″E	1,180	NW	7.66	232	268	149	197	52.6
9a	Hala Długa Glade (g)	49°32′47″N 20°07′55″E	1,200	SE	3.44	69	74	61	67	
9b	Hala Długa Glade (g)	49°32′42″N 20°07′46″E	1,200	SE	4.52	160	38	16	63	
21	Turbaczyk (f)	49°34′44″N 20°06′55″E	1,050	NW	7.14	201	132	9	96	87.7
22	Konina Valley (f)	49°34′49″N 20°08′01″E	680	NW	17.1	372	332	208	286	32.9
23	Mostownica (f)	49°33′52″N 20°07′55″E	950	SE	3.16	133	25	13	46	
24	Lubomierz (f)	49°35′31″N 20°10′08″E	840	NW	8.65	343	60	6	105	
25	Kosarzysko Glade (f)	49°34′45″N 20°10′13″E	1,020	SE	3.64	165	56	7	51	
26	Kudłoń (f)	49°34′18″N 20°10′35″E	1,250	SE	6.07	294	250	117	195	
27	Podskale (f)	49°35′04″N 20°11′33″E	970	NW	11.9	140	695	312	333	27.7
29	Kiczora Kamien. (f)	49°36′01″N 20°15′18″E	850	SE	4.68	296	56	11	77	36.1
29a	Kiczora Kamien. (g)	49°35′53″N 20°15′29″E	960	SE	4.90	178	77	39	77	
30	Szczawa Bukówka (f)	49°37′28″N 20°16′23″E	600	SE	3.42	146	55	18	56	
42	Jaworzyna Kamien. (f)	49°32′52″N 20°09′48″E	1,250	SE	5.28	347	235	147	205	
43	Kamienica Valley (f)	49°33′44″N 20°11′15″E	970	SE	2.47	42	38	33	36	50.7
45	Jaszcze Duże (f)	49°32′11″N 20°12′01″E	800	SE	2.25	87	23	6	30	
47	Skałka (f)	49°33′23″N 20°13′43″E	1,050	SE	5.94	428	203	14	109	
48	Gorc (f)	49°33′53″N 20°14′53″E	1,110	SE	3.10	314	106	27	81	73.1
49	Gorcowe (f)	49°32′45″N 20°16′14″E	750	SE	7.27	349	83	33	113	
61	Bukowina Obidow. (f)	49°32′00″N 20°02′31″E	980	NW	9.98	382	433	396	403	49.3
62	Lepetnica Valley (f)	49°32′23″N 20°02′47″E	830	NW	4.44	152	252	111	161	
63	Bukowina Miejska (g)	49°31′57″N 20°04′42″E	1,110	SE	4.93	271	199	9	122	
64	Bukowina Waksm. (f)	49°31′25″N 20°05′56″E	1,050	SE	4.20	333	62	5	68	
65	Mały Kowaniec (f)	49°30′38″N 20°03′47″E	780	SE	3.08	161	107	12	35	41.4
66	Jankówki (f)	49°31′33″N 20°08′14″E	1,050	SE	7.39	287	394	50	204	45.8
68	Łopuszna/valley (f)	49°30′26″N 20°07′50″E	730	SE	2.18	72	156	99	106	27.2
69	Forędówki (f)	49°31′05″N 20°11′36″E	870	SE	2.78	84	30	31	51	54.9
80	Knurowska Pass (f)	49°29′50″N 20°10′46″E	800	SE	6.17	379	192	35	124	
81	Studzionki (f)	49°29′41″N 20°12′60″E	890	SE	3.58	111	202	277	200	
82	Szlembark (f)	49°29′19″N 20°12′48″E	780	SE	4.61	268	119	12	74	64.6
83	Runek (f)	49°29′52″N 20°15′49″E	980	SE	6.21	199	41	8	73	31.7
84	Morgi Glade (f)	49°29′31″N 20°18′00″E	1,000	SE	2.34	49	52	32	42	
85	Mizerna/valley (f)	49°28′43″N 20°17′46″E	760	SE	4.31	187	100	43	91	13.4
87	Lubanskie (f)	49°30′35″N 20°20′14″E	640	SE	4.51	187	45	15	70	
88	Lubań (f)	49°29′22″N 20°20′29″E	1,180	SE	7.59	361	288	95	198	45.4
88a	Lubań (g)	49°29′18″N 20°20′29″E	1,190	SE	4.49	338	48	3	68	
89	Kotelnica (f)	49°27′47″N 20°23′37″E	660	SE	3.15	49	39	35	39	69.8
91	Ochotnica Stasichy (f)	49°30′14″N 20°14′31″E	730	SE	6.40	285	184	60	130	
92	Ochotnica Kudows. (f)	49°30′22″N 20°18′29″E	640	SE	3.69	151	185	33	84	
93	Ochotnica Ligasy (f)	49°30′42″N 20°22′35″E	620	SE	2.86	52	52	40	45	
94	Tylmanowa Ziem. (f)	49°28′58″N 20°23′40″E	530	SE	4.93	236	78	18	76	

^a^(f)—forest, (g)—glade; *Kamien.*—Kamieniecka, *Obidow.*—Obidowska, *Waksm.*—Waksmundzka, *Ochotn.* —Ochotnickie, *Kudows.*—Kudowskie, *Ziem.*—Ziemianki

^b^Uncertainty of the site location identification amounts to 80 m

^c^Two contaminated Gorce Mts. parts distinguished by the authors: *NW* north-western part, *SE* south-eastern part

^d^Relative uncertainty of less than 10 %; uncertainty of core height was included

^e^Activity concentration in 1st, 2nd, 3rd soil layer and in a 10 cm soil layer (whole core) respectively; relative uncertainty less than 5 %

^f^Relative uncertainty less than 10 %

### Vertical distribution of Cs-137

As the result of measurements, Cs-137 activity concentration was determined in individual soil layers, and then the vertical distribution of Cs-137 (depth profile). Statistical parameters for each of the three layers are presented in Table 2; due to the different Cs-137 deposition in the Gorce Mts. (cf. next chapter), the data is presented separately for the two distinguished parts of the study area. The values presented in Table 2 only provide information on the mean concentrations in individual layers, thus they should not be the used as a basis for estimation of concentration variability in soil profile.

**Table 2:** Cs-137 activity concentration in soil layers

Layer	Range	Mean/median	Standard deviation	CV
Gorce Mts.—north-western part^a^				
	(Bq/kg)	(Bq/kg)	(Bq/kg)	(%)
1. layer^b^	85–533	286/259	122	43
2. layer^b^	60–947	405/332	283	70
3. layer^b^	6–412	154/111	133	86
Mean^c^	87–540	253/210	148	59

Gorce Mts.—south-eastern part^a^				
	(Bq/kg)	(Bq/kg)	(Bq/kg)	(%)
1. layer^b^	42–428	207/187	113	54
2. layer^b^	23–395	115/78	89	78
3. layer^b^	3–277	43/31	53	125
Mean^c^	30–205	91/75	53	58

Gorce Mts.—whole area^a^				
	(%)	(%)	(%)	(%)
1. layer^d^	8–81	39/33	21	55
2. layer^d^	16–77	38/33	14	37
3. layer^d^	2–64	23/18	16	70

^a^Number of samples: NW Gorce Mts. 15, SE Gorce Mts. 34, Gorce Mts.—whole area 49

^b^Cs-137 activity concentration in a successive soil layer

^c^Mean activity concentration in a 10 cm soil layer

^d^Percentage of a successive soil layer in Cs-137 total activity in 10 cm soil layer

The change of Cs-137 activity concentration in soil profile is not uniform. In the entire Gorce Mts., the profiles, whose concentration rapidly falls with depth (69 %) dominate, there are profiles in which the maximum concentration appears in the second layer (16 %), and also profiles in which concentration is more or less constant in all the layers (10 %).

The total activity of Cs-137 (Bq) in individual layers was calculated based on the Cs-137 activity concentrations in individual soil layers (Bq/kg); results show that, on average, in the Gorce: in the first layer there is approx. 39 % of Cs-137 deposited per 10 cm soil layer, in the second 38 %, and in the third 23 %.

### Deposition of Cs-137

Based on the analysis of deposition of Cs-137 in the selected sampling points, the authors distinguished two areas of the Gorce Mts. (cf. Fig. 1; Tables 1, 3): south-eastern and north-western; one has double the caesium contamination of the other. The deposition in the north-western part, which has a higher level of contamination, is in the range 4.5-17.1 kBq/m^2^ with an average of 9.9 kBq/m^2^, while in the less contaminated south-eastern part deposition ranges between 2.2 and 7.6 kBq/m^2^ with an average of 4.4 kBq/m^3^. The border of the two parts runs approximately along the line Nowy Targ—Mt. Turbacz—Lubomierz (cf. Fig. 1).

**Table 3:** Deposition of Cs-137 and Cs-137 activity concentration in *H*. *physodes* in the Gorce Mts.

Area	Range	Average/median	Standard deviation	CV
Cs-137 deposition	(kBq/m^2^)	(kBq/m^2^)	(kBq/m^2^)	(%)
Gorce Mts.—north-western part^a^	4.5–17.1	9.9/9.7	3.6	36
Gorce Mts.—south-eastern part^a^	2.2–7.6	4.4/4.4	1.5	35
Gorce Mts.—whole area^a^	2.2–17.1	6.1/4.9	3.5	57
Mt. Mostownica “local variability”^a^	2.2–8.2	4.8/3.9	1.9	40

*Hypogymnia physodes*	(Bq/kg)	(Bq/kg)	(Bq/kg)	(%)
Gorce Mts.^b^	13–88	47/45	18	38

^a^Number of samples: NW Gorce Mts. 15, SE Gorce Mts. 34, Gorce Mts.—whole area 49, Mt. Mostownica 19

^b^Number of samples: 25

It seems that the reason for such significant differences in contamination in the two distinguished parts may be possibly due to the various meteorological conditions when radioactive contamination (radioactive cloud) from the Chernobyl disaster spread in the atmosphere. However, the Polish Institute of Meteorology and Water Management (IMGW) data indicates that the impact of rainfall differences should probably be excluded. It was found that in the period when the “Chernobyl cloud” was spreading over Southern Poland, i.e., between 28th April and mid-May 1986, the rainfall in the Gorce Mts. area was minimal (up to fifteen or so mm daily; mainly on 30th April and between 8th May and 11th May) and no differences in the rainfall level between the two parts of the Gorce Mts. were identified.

**Fig. 2:**
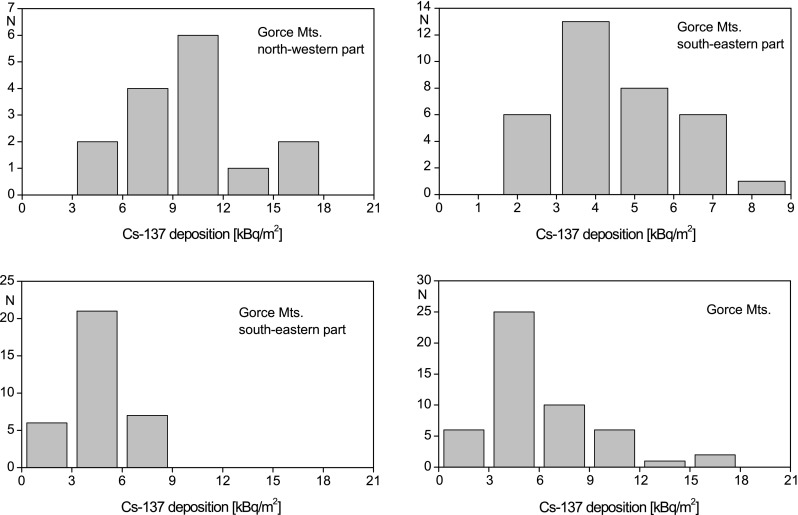
Statistical distribution of Cs-137 deposition in the Gorce Mts. The distribution of deposition values in the Gorce Mts. SE part has been presented twice: once at the bin width being the same as in the NW part (for comparison of the two parts), and again at the bin width being half of that in the NW part

The statistical distribution of the Cs-137 deposition in the whole Gorce Mts. and in the two distinguished parts is presented in Fig. 2. As data is scarce, it is difficult to determine the nature of these distributions. Nevertheless, it is characteristic that in each of the two distinguished parts of the Gorce Mts. the average and median values of deposition are almost equal. These statistical parameters differ significantly in log-normal distribution generally.

**Fig. 3:**
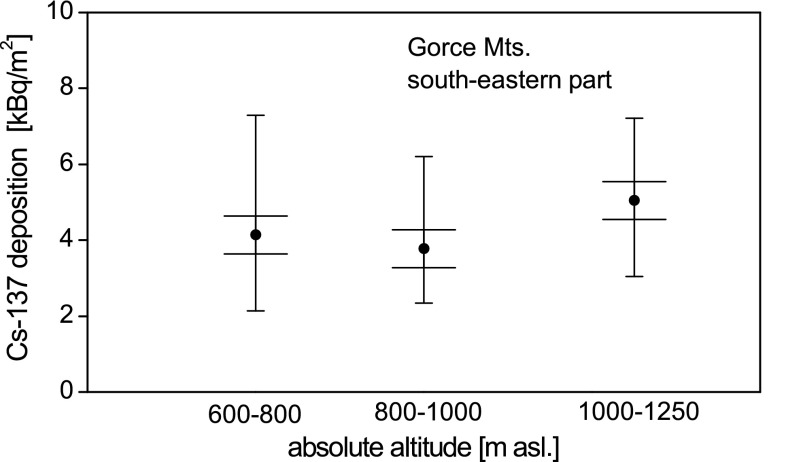
Dependence between the Cs-137 deposition and the altitude of the sampling site. Each entry shows: the mean value and its uncertainty (*longer horizontal line*) and variability range (*shorter horizontal line*)

The relationship between the Cs-137 deposition values and the absolute altitude of the sampling sites is shown for the south-eastern part of the study area (Fig. 3) as more data was available just for this part of the Gorce Mts. The analysis of the graphs indicates that there is no basis to assume any dependence between Cs-137 deposition and the absolute altitude.

In order to compare the results obtained with those of other authors, the literature data has been decay-corrected to 1st July 2005. According to Mietelski et al. [10], the Gorce Mts. are situated on the border between the deposition areas 1.5–4.4 and 4.4–7.3 kBq/m^2^. The Radioecological Map of Poland [11] elaborated using the in situ method assigns the Gorce Mts. to the area with a deposition of 0–5.9 kBq/m^2^, while the average deposition in the then Nowy Sącz Voivodeship that encompassed the area of the Gorce Mts. was 2.5 kBq/m^2^. In 2006 the Central Laboratory for Radiological Protection (CLOR) carried out measurements in five villages situated in the Gorce Mts. [12]. The deposition of Cs-137 ranged between 0.7 and 2.0 kBq/m^2^, while the average deposition in the Małopolska Voivodeship encompassing at that time the Gorce Mts. was around 3.8 kBq/m^2^. Kubica et al. [13] measured deposition in the Tatra Mts. (located approx. 30 km from Gorce Mts.) and obtained results between 0.2 and 17.5 kBq/m^2^ with an average of 5.6 kBq/m^2^. Comparison of the results obtained with the previous data is justified because the Cs-137 deposition decreases only slightly faster than Cs-137 decays (Cs-137 T_1/2_ = 30 years).

The average deposition of 9.9 kBq/m^2^ measured by the present authors in the north-western part of the Gorce Mts. is significantly higher than the values presented on the maps of Cs-137 deposition in Poland [10–12]. This difference is probably mainly due to averaging the local values on these maps as the maps were constructed to reflect regional variations. Therefore, they do not show small areas with a higher deposition of Cs-137.

**Fig. 4:**
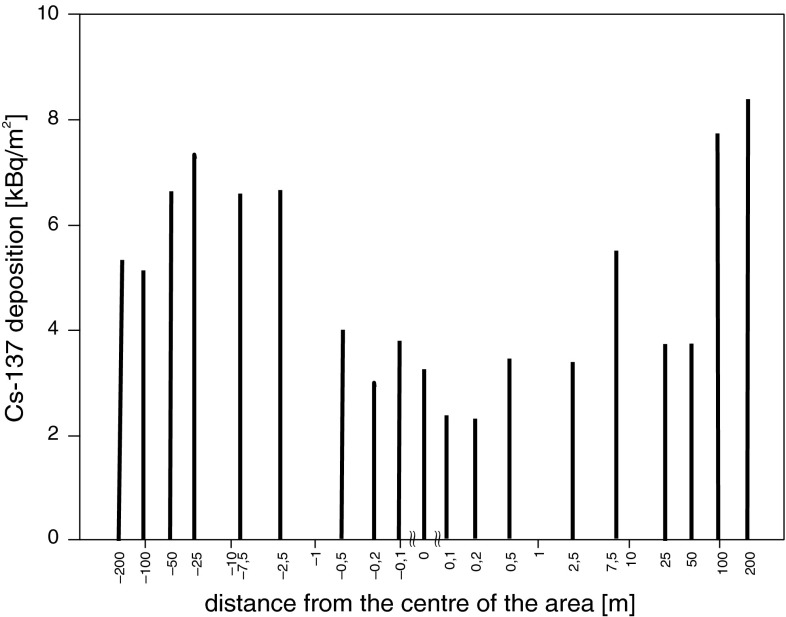
Cs-137 deposition value in 19 sampling points located on 400 m long slope profile of Mt. Mostownica massif. Values on the x-axis represent the distance of a sampling point from the centre of the investigated area; the “+” and “−” symbols, indicate that the point is located accordingly above or below the centre of the area

When interpreting the Cs-137 deposition data, its significant local variability should be taken into account. It is defined as the coefficient of variation (CV) of statistical distribution of the deposition in the case of multiple sampling points around one site. The author determined local variability of Cs-137 deposition in 19 points on the 400 m slope located in the Mt. Mostownica massif, in the south-eastern part of the Gorce Mts. (cf. Fig. 4 and Table 3). The deposition value varied in the interval 2.5–9.2 kBq/m^2^, the mean value was 5.3 kBq/m^2^, and CV 40 %. The CV value is slightly above the variability interval of the literature data oscillating (for an area of approximately 0.1–1 km) in the range 10–30 % (cf. juxtaposition in [14] and [15–16]). The CV value slightly higher than quoted by other authors, can be explained by the undulations of the Gorce Mts. area and the possibility of Cs-137 accumulation in surface irregularities of the area; as a result, there are points of significantly higher deposition and the CV value increases. An analysis of the data presented in Table 3 indicates that the variability in the north-western and south-eastern parts of the Gorce Mts. (ca. 35 %) is comparable with the local variability (40 %). Therefore, it may be assumed that each of the two parts is contaminated evenly, and the spreads of deposition values obtained in these parts for various sampling points (cf. Fig. 1), are associated with the local variability of the deposition.

### Lichens

The results of measurements of Cs-137 activity concentration in *H*. *physodes* from selected sites in the Gorce Mts. are presented in Table 3. The Cs-137 activity concentration in lichens is in the range 13–88 Bq/kg d.m. (dry mass) with an average of 47 Bq/kg d.m. It is characteristic that the average and median values are almost equal.

The Cs-137 activity concentration values measured are comparable to the literature data of around 100 Bq/kg d.m. for samples of *Pseudevernia furfuracea* and *Parmeliaceae* family from South and South-Eastern Poland [17, 18].

The local variability (cf. [19]) has been estimated in the sampling site (an area of approx. 1 ha) located in the forest of the Mt. Turbacz (site no 9), where lichen samples were collected from ten trees (five beech and five spruce specimens). The variability is approx. 50 % being comparable to the variability established over the whole of the Gorce Mts. area (CV = 38 %). Therefore, it may be accepted that the spreads of Cs-137 activity concentrations in lichens, obtained for various sampling points in the Gorce Mts., are associated with the local variability.

In the opinion of the present authors, one of the reasons for the high local variability of the Cs-137 activity concentration in the lichen is the growth of the lichen resulting in two co-occurring types of the thallus in a single sample:

- the thallus highly contaminated shortly after the Chernobyl disaster in 1986 (direct contamination) or the thallus contaminated by the former due to Cs-137 leaching (indirect contamination),
- the thallus formed much later, i.e., in the period when the activity concentration of Cs-137 in the air was lower by several orders of magnitude.

To sum up, the Cs-137 activity concentration in the lichen is currently due first of all to the contribution of the thallus contaminated shortly after the Chernobyl disaster, to the total thallus volume sampled. This factor probably significantly differs from one sample to another.

## Conclusions

In the relatively small area of the Gorce Mts. (several hundred km^2^), the authors established in detail the activity concentration of Cs-137 and its spatial distribution in soils and lichen. Such investigations provide, on the one hand, information on the current distribution of the caesium contamination, and on the other hand, may establish a point of reference in future changes of this distribution in the course of time.

Two parts of the Gorce Mts. have been distinguished on the basis of different deposition of Cs-137. The respective average values are 4.4 and 9.9 kBq/m^3^ as at 1st July 2005, providing a relative difference of around 2. The value 9.9 kBq/m^2^ is much higher than the values given in the maps of Cs-137 deposition in Poland.

The average Cs-137 activity concentration in the *H*. *physodes* from the Gorce Mts. is 47 Bq/kg d.m.

When interpreting this data, significant local variability of measured quantities (Cs-137 deposition, Cs-137 activity concentration in lichen) should be taken into account. This local variability amounts to several dozen percent and is comparable with the variability calculated for the whole area of the Gorce Mts. (for deposition, for the distinguished two Gorce Mts. parts, respectively). Thus, there are no grounds to draw isolines of measured quantities on the map of the Gorce Mts.
